# CHCHD2: The Power House's Potential Prognostic Factor for Cancer?

**DOI:** 10.3389/fcell.2020.620816

**Published:** 2021-01-12

**Authors:** Rohit Gundamaraju, Wenying Lu, Rishya Manikam

**Affiliations:** ^1^ER stress & Gut Mucosal Immunology Group, School of Health Sciences, University of Tasmania, Launceston, TAS, Australia; ^2^Respiratory Translational Research Group, Department of Laboratory Medicine, School of Health Sciences, University of Tasmania, Launceston, TAS, Australia; ^3^Emergency and Acute Care Centre, Faculty of Medicine, University Malaya, Kuala Lumpur, Malaysia

**Keywords:** CHCHD2, mitochondria, cell migration, metastasis, prognosis, OXPHOS

## Introduction

Coiled-coil-helix-coiled-coil-helix domain-containing protein 2 (CHCHD2) is a mitochondrial resident protein which is relatively novel. Variants of CHCHD2 have been linked to Parkinson's disease and CHCHD2 is reportedly found to possess role of transcription factor (Liu et al., 2020). CHCHD2 is a mixed blessing. CHCHD2 loss can lead to impaired mitochondrial respiration and energy production, but on the other hand it is reported to inhibit apoptosis through regulating Bax activation (Liu et al., 2015). Hence, it has been recently spotted as a therapeutic target in cancer. However, its complete mechanistic role in cancer remains elusive. Hence, we wish to set forth its role as a potential prognostic marker for cancer.

## CHCHD2 and its Attainable Potential

Novel data suggests that CHCHD2 serves as modulator for scavenging ROS (reactive oxygen species) and activation of BCL-XL. The functional role of CHCHD2 in inhibiting apoptosis is intriguing. It is well-understood that the mitochondrial outer membrane permeabilization (MOMP) controls apoptosis. MOMP is controlled by proteins like Bax. During some stressed conditions, Bax is in turn regulated by anti-apoptotic proteins like Bcl-xL. CHCHD2 binds to Bcl-xL and inhibits the mitochondrial accumulation and oligomerization of Bax which exemplifies the vital role of CHCHD2 in regulating cell death (Liu et al., 2015). We have recently discovered the correlation between endoplasmic reticular stress (ERS) and CHCHD2 in our study (Wilson et al., 2020). Succeeding our research question of mysterious survival of colonic goblet cells despite of severe ERS in Winnie mice (murine model of chronic ERS and spontaneous colitis mouse model) (Heazlewood et al., 2008), we have performed a proteomic study on the isolated goblet cells (GC's). We have established a partial correlation between ERS and CHCHD2. We discovered alterations in the goblet cellular proteome in Winnie mouse. There were profound effects across mitochondria and ER. CHCHD2 when focused in the verge of identifying the righteous candidate that might promote the survival of GC's despite their chronic state of ER stress. The results of our proteomics analysis were consistent with elevated ROS production as a consequence of ER and mitochondrial stress in GC's. Our outcome supports the evidence of capability of CHCHD2 in ROS-dependent translocation from the mitochondria to the nucleus and transactivation of genes involved in mitochondrial respiration (Liu and Zhang, 2015). Our immune fluorescence results also signified that CHCHD2 was found in both ER and mitochondria denoting a linkage. In addition, CHCHD2 despite of acting as a potential negative regulator of mitochondrial apoptosis might also be a part of a feedback mechanism that further stimulates oxidative phosphorylation and survival of Winnie GCs (Wilson et al., 2020). CHCHD2 is essential for mitochondrial integrity. The pro-survival ability of CHCHD2 was proved in the case of mice and Drosophilia where CHCHD2 loss leads to ROS-dependent apoptosisvia destabilization of cytochrome c (Liu et al., 2015, Meng et al., 2017). CHCHD2 also possesses role attenuation of ROS generation and, importantly, the inhibition of the intrinsic apoptosis pathway under ERS and enhances complex IV activity by stimulating COX4I2 expression (Aras et al., 2013). Hence, this might be the reason for the apoptosis evasion by the GC's under ERS in our study (Wilson et al., 2020).

## Mitochondria, the Next Big Cancer Drug Target?

Apart from being indispensible for energy production and survival support, mitochondria is a crucial controller of intrinsic cell death pathway. Pharmacological drugs have been utilized to impinge on mitochondrial membrane permeabilization since it constitutes a central event during mitochondrial apoptosis. Class of drugs include modulators of the B-cell lymphoma protein 2 (BCL-2) protein family, metabolic inhibitors, voltage-dependent anion channel (VDAC)-targeting and adenine nucleotide translocase (ANT)-targeting agents, redox-active molecules, retinoids, heat-shock protein 90 (HSP90) inhibitors, as well as natural compounds with distinct mechanisms of action (Roth et al., 2020). Mitochondria orchestrate various mechanisms of programmed cell death by controlling processes like translocation of pro-apoptotic proteins from the mitochondrial intermembrane space to the cytosol (Fulda et al., 2010). Furthermore, mitochondria play a major role in multiple forms of non-apoptotic cell death and, in particular, in necroptosis (regulated necrosis). As mitochondrial functions are often altered in neoplasia, mitochondrially-targeted compounds represent a promising approach to eradicate chemotherapy-refractory cancer cells (Fulda et al., 2010, Roth et al., 2020).

The fact that mitochondrial malfunction results in tumorigenesis is proven by responses like chromosomal instability to stimulus mutations of the mitochondrial or nuclear DNA that affect components of the mitochondrial respiratory chain result in inefficient ATP production, ROS overproduction and oxidative damage to mitochondria and other macromolecules (Modica-Napolitano and Singh, 2004). Polymorphisms and mutations of DNA also can be interrelated to multiple malignancies. Mitochondrial dysfunction has been correlated to apoptotic dysfunction, reduced autophagy, and uncontrollable proliferation (Galluzzi et al., 2010, Kroemer and Pouyssegur, 2008).

Mitochondrial targeting drugs can be put into use by utilizing mechanisms like: (1) mitochondrial permeability where chemical inhibitors are used to act on permeability transition pore complex to induce programmed cell death, (2) targeting mitochondrial outer membrane permeabilization, (3) targeting metabolism, which can induce apoptosis by reversing cellular activities like hyperglycolysis, and last but not the least (4) targeting heat shock class of proteins like HSP90 since in cancer cells the HSP90–TRAP1 complex reportedly modulates cyclophilin D (CYPD) regulated mitochondrial permeability transition pore (MPT) via protein folding mechanisms (Kang et al., 2007). Mitochondrially-targeted HSP90 antagonists might therefore be exploited to interfere with signaling networks in specific subcellular compartments of tumourcells (Fulda et al., 2010, Kang et al., 2007).

## Mitochondrial OXPHOS and Course of Linkage with CHCHD2

The evolution of molecular biology, oncogenes and tumor suppressor genes in recent years shifted the general interest in the cancer field into directions other than metabolism and promoted the Warburg hypothesis or its consideration as an epiphenomenon of cell transformation. The revival of importance of mitochondria in biological studies has happened due to the recognition of its key role in execution of apoptosis and participation in a wide array of diseases like cancer. It has become increasingly clear that cancer is associated with a decrease in the activity and expression of β-F1-ATPase, a key subunit of the mitochondrial ATP synthase. This modulation has been evidenced to limit oxidative phosphorylation and to trigger the induction of glycolysis to provide energy to the cell thereby aligning the Warburg effect as a salient hallmark of the cancer (Ristow and Cuezva, 2009). In cancers, these energy pathways are activated in response to oncogenic signaling. Cancer cells become distinct where they can successfully utilize glycolysis and oxidative phosphorylation (OXPHOS) which can facilitate metastasis and resistance (Jia et al., 2018). Mitochondrial oxidative phosphorylation (OXPHOS) is the principal cell's generator of ATP to energy homeostasis and human health (Baughman et al., 2009). Five protein complexes that are formed from ~90 distinct protein subunits comprise OXPHOS, which occupy in the inner mitochondrial membrane (Baughman et al., 2009). Mitochondrial dysfunction is known to be associated with aging, age-related diseases, and cancer (Kudryavtseva et al., 2016). Mitochondrial biogenesis and respiration are active aspects during metastasis, and the inhibition of OXPHOS suppresses metastasis in breast and cervical cancer, which indicates that the hybrid glycolysis/OXPHOS phenotype might be associated with the increased metastatic potentials (Jia et al., 2018).

**Figure 1:**
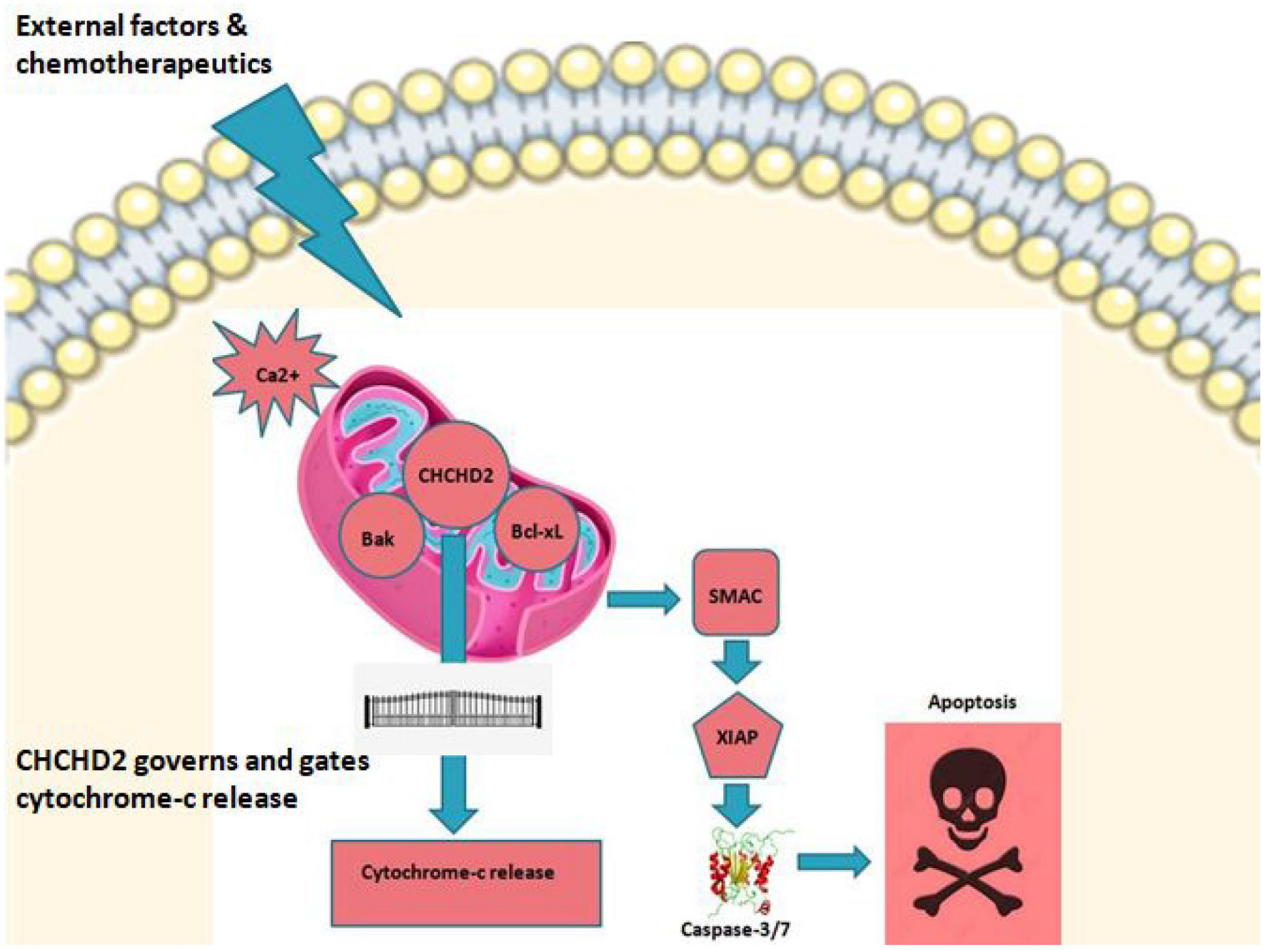
CHCHD2 and role play. CHCHD2 in mitochondria under stress or under external stimulus controls apoptosis in cancer. Among the numerous proteins and protein complexes that control apoptotic pathways, IAP (inhibition of apoptosis) proteins play an important role. There are two predominant ways by which IAP proteins regulate apoptosis. c-IAP proteins do not bind caspases at a physiologically meaningful level. Instead, they regulate caspase activation indirectly through their E3 ligase activity and modulation of TNF-mediated cell death as well as Toll signaling, innate immunity and NF-κB pathways. Anti-apoptotic activity of XIAP stems primarily from its direct binding to and inhibition of caspase-3,−7, and−9. On the other hand, IAP's are negatively regulated by IAP-antagonists like second mitochondrial derived activator of caspases (SMAC). Upon triggering of intrinsic pathway, SMAC is released into cytosol where caspase activation is initiated leading to programmed cell death. It is believed that CHCHD2 drives this principal cell fate interplay thereby acting as a vital switch in regulating mitochondrial mediated apoptosis.

Mitochondrial dysfunction caused by the mutations or rearrangements of mitochondrial DNA (mtDNA) is related to the OXPHOS dysfunction (Kudryavtseva et al., 2016). The crosstalk between mitochondrial dysfunction and cancer progression has been related to the mtDNA mutations or rearrangements in a variety of cancers (Deng and Haynes, 2017). Most of these mutations cause the reduction of OXPHOS efficiency (Singh et al., 2009) and drive cancer cells to depend more on glycolysis for ATP production. It also aids for functions like repression of mitochondrial apoptosis, growth and survival (Deng and Haynes, 2017). CHCHD2 promotes oxygen consumption of mitochondria and consistently co-expressed with OXPHOS subunits (Baughman et al., 2009). CHCHD2 assists to maintain OXPHOS by binding to cytochrome c oxidase (COX) or BCL2-like 1 isoform 1(Bcl-xl) (Liu et al., 2015). CHCHD2 binds to cytochrome c along alongside Bax inhibitor-1 superfamily, and modulates cell death signaling, suggesting that CHCHD2 essentially regulates the functions of cytochrome c in both OXPHOS and cell death in response to mitochondrial stress (Meng et al., 2017). These internal mechanisms like mitochondrial-tumor microenvironment crosstalk can be useful immensely in understanding therapeutics (Roth et al., 2020) some of which are illustrated in Figure 1. Moreover, OXPHOS has been greatly correlated with cancer spread. Increased mitochondrial OXPHOS has been linked to increased proliferation and inducing cancer stemness (Zacksenhaus et al., 2017).

## Role of CHCHD2 in Cancer Progression

CHCHD2 over expression has been implicated in diverse cancers. CHCHD2 was not only recorded in hepatocellular carcinoma (HCC) cells but also tissues whereCHCHD2 protein level was remarkably relatable with poor differentiation, lymph node metastasis, local tissue invasion, angiogenesis, and poor prognosis in patients (Yao et al., 2019). CHCHD2 silencing in HCC cells lead to increased programmed cell death denoting the role of CHCHD2 in mitochondrial mediated cell death. Additionally, CHCHD2 knockdown led to down regulation of CD105 which is a noted marker for angiogenesis which now exposes the role of CHCHD2 in tumor angiogenesis (Yao et al., 2019). CHCHD2 expression is also increased in breast cancer. CHCHD2 levels are correlated with increased metastasis and poor prognosis in breast cancer patients. There was a marked lessening of cell migration and proliferation noted when CHCHD2 was knocked out in MCF-7/DTX and SKBR3/DTX cells (Ma et al., 2020). Surprisingly, MMP2 expression was positively co relatable to CHCHD2 expression in the same study. The role of CHCHD2 was clearly established in stimulating proliferative and migratory potentials in Docetaxel-resistant breast cancer cells by upregulating MMP2 (Ma et al., 2020). Mitochondrial mediated therapeutics in cancer can be achieved using various pathways and targetting inside mitochondria including ketone bodies and L-lactate is one amongst. In a study, a MCT1/2 inhibitor (AR-C155858) was employed to impede the functioning of the above sources, ketone bodies and L-lactate (Lamb et al., 2014). These findings showcase that AR-C155858 treatment markedly reduced the mammospheres formation of MCF7 and T47D cell lines. CHCHD2 in this study was the highest up-regulated genes with a fold up-regulation of 5.79 in both MCF7 and T47D cells. Additionally, it was reported that CHCHD2 was the most transcriptionally up-regulated protein which warrants the key role of CHCHD2 stem cell metabolism and cell migration (Lamb et al., 2014). CHCHD2 was attendant even in invasive ductal carcinoma where the expression of CHCHD2 was recorded twice in malignant tissue compared to benign tissue and about 18 times higher in breast cancer cell lines compared to control cells thereby suggesting its association with invasive metastasis (Aras et al., 2019).

Majority of lung cancers are reported to be non-small cell lung carcinoma (NSCLC) with high recurrence. CHCHD2 was found highly expressed in NSCLC especially in NIH3T3 fibroblasts and specimens (Seo et al., 2010, Song et al., 2015). The association between CHCHD2 and clinicopathological features revealed that patients exhibited shorted survival time. Furthermore, CHCHD2 was co-expressed with HIF-1a in NSCLC. A faint expression of CHCHD2 and HIF-1a had an average mean survival time of about 50 months, whereas a high expression of CHCHD2 and HIF-1a showed an average mean survival time of about 17 months (Yin et al., 2020). The same study also elucidated the relation between tumor size and CHCHD2 expression, staging, and survival rate. This clearly describes that the grade of differentiation, lymph node metastasis, CHCHD2 and HIF-1α expressions were all present in significantly higher hazard ratios, which indicated that these factors would been independent prognostic factors of NSCLC(Yin et al., 2020). CHCHD2 is also co-amplified with EGFR in patient derived xenografts compared to normal lung (Wei et al., 2015). Alteration in expression of CHCHD2 markedly reduced the proliferation rate in LPC43 cells (Wei et al., 2015). CHCHD2 expression was significantly escalated in renal cell carcinoma (RCC) tissues, where it was notified that CHCHD2 expression was increased in stage II-IV of the disease. CHCHD2 knockdown strikingly decreased the migration, proliferation and angiogenesis in RCC cells. Further, CHCHD2 knockdown in 786-O cells led to a demonstrated decrease in expression of MMP-2 and VEGF (Cheng et al., 2019). This clearly states that CHCHD2 is actively involved in angiogenesis of RCC.

## Future Directions and Conclusion

The most important point in the drug discovery resides in the fact that mitochondria are widely involved in various mechanisms and pathways. CHCHD2 engages in mitochondrial and extra-mitochondrial protein interactions and is a positive regulator for cell proliferation and migration. Its co-amplification and promotion of oncogenes suggests its role as a novel molecular marker and a potential target in the cancer therapy. CHCHD2 drives OXPHOS through regulation of Cyt c and crista integrity. Finally, CHCHD2 triggers OXPHOS ensuing in increased anabolism, stemness which leads to increased proliferation and metastasis. Therefore, CHCHD2 must be further studied since profound insights into the immune-metabolic and cytogenic pathways might culminate in the design of combination regimens for multiple cancers.

## Author Contributions

RG and WL drafted the manuscript. RM reviewed and approved the manuscript. All authors contributed to the article and approved the submitted version.

## Conflict of Interest

The authors declare that the research was conducted in the absence of any commercial or financial relationships that could be construed as a potential conflict of interest.
